# Unilateral Spatial Neglect After Stroke: A Pragmatic Approach to Assessment and Rehabilitation

**DOI:** 10.3390/jcm15156064

**Published:** 2026-08-04

**Authors:** Giulia Salti, Benedetta Formelli, Benedetta Piccardi, Eleonora Barucci, Anna Poggesi

**Affiliations:** 1 Stroke Unit, Careggi University Hospital, 50134 Florence, Italy; 2 NEUROFARBA Department, Neuroscience Section, University of Florence, 50134 Florence, Italy; 3 Emergency Neurology Unit, Careggi University Hospital, 50134 Florence, Italy

**Keywords:** stroke, unilateral spatial neglect, unilateral spatial neglect assessment, post-stroke rehabilitation, cognitive disorders, unilateral spatial neglect cognitive rehabilitation

## Abstract

Unilateral spatial neglect (USN) is a neuropsychological syndrome characterized by a reduced awareness of, and attention toward, stimuli located in the contralesional space. The left side of the space is most frequently affected, due to the higher incidence of the disorder following right-hemisphere lesions, particularly within the territory of the right middle cerebral artery and, less commonly, the right anterior cerebral artery. Although USN can also occur following left-hemisphere damage, it is typically less severe and tends to resolve more quickly. USN is a common consequence of acquired brain injury, especially of vascular origin, and significantly impairs a patient’s functional abilities and quality of life. Its high incidence in the acute phase after stroke, together with its potential persistence into the chronic stage, makes early diagnosis and timely rehabilitation essential. In this narrative review, we provide an overview of current assessment tools and rehabilitation approaches for post-stroke USN, with a focus also on recent advances and emerging therapeutic strategies. To illustrate diagnostic and therapeutic decision making, a representative case of a patient admitted to a Stroke Unit is reported, illustrating the application of assessment methods in a real-world setting. The aim of this review is to offer clinicians practical tools and evidence-based strategies to support a more effective and individualized management of patients affected by this complex and disabling condition.

## 1. Introduction

Unilateral spatial neglect (USN) is a neuropsychological disorder characterized by a reduced awareness of and attention to stimuli in the contralesional hemispace, as well as by difficulties in initiating movements toward that side of the space [1,2,3] or in using the contralateral limbs [4]. Patients are unable to detect, attend to, or respond to meaningful stimuli located in the contralesional hemispace. This syndrome may coexist with sensory and motor deficits, but it cannot be explained solely by them [5,6]. In fact, USN may occur alongside deficits such as hemiplegia or visual impairments, but it can also be observed independently of these conditions [7].

USN can be found in patients with brain injuries involving either hemisphere, but it is more frequent, severe, and disabling following right-hemisphere lesions [8,9]. While this syndrome may result from various etiologies, vascular lesions are the most common cause [4]. The occurrence of USN varies across studies, from 20% to 80%, depending on brain lesion locations, time of assessment after stroke onset, and assessment methods. In terms of distribution, this syndrome is found in 38% of individuals with right-hemisphere damage and in 18% of those with left-hemisphere damage. Regarding its temporal evolution, approximately 45% of right brain stroke patients exhibit USN in the acute phase (within the first week), 40% in the subacute phase (1 week to 3 months), 29% in the post-acute phase (6 to 12 months), and 20% in the chronic phase (beyond 1 year) [2].

Critically, USN is a strong predictor of poor functional outcomes [10,11,12,13]. In fact, it is associated with more severe strokes, longer hospital stays, greater disability and dependence at discharge, increased mortality, and a higher likelihood of discharge to institutional care. Despite these consequences, USN is often under-recognized in the early stages and is not treated with a level of therapeutic intensity that reflects its clinical impact [14]. Given the incidence of this syndrome and its impact on the recovery of other functions, such as motor and functional abilities, it would be important to identify and treat it with a specific training to support overall rehabilitation outcomes [15].

USN is a syndrome that has gained significant scientific attention, with a growing number of reviews published on the subject, particularly in the last decade. This narrative review aims to provide clinicians and rehabilitation professionals with an updated overview of post-stroke neglect, focusing on the clinical symptoms and neuropsychological features of affected patients, current assessment tools, and non-pharmacological treatment approaches. A clinical case will also be presented to illustrate the practical implications and outcomes in the acute and sub-acute post-stroke phase.

## 2. Methods

This narrative review was based on a non-systematic literature search conducted using PubMed and Google Scholar. The following keywords were used, alone or in combination: “Unilateral Spatial Neglect”, “Post-stroke Unilateral Spatial Neglect”, “Anatomical correlates of Unilateral Spatial Neglect”, “Assessment of Unilateral Spatial Neglect”, “Rehabilitation of Unilateral Spatial Neglect.”, “Cognitive rehabilitation for Unilateral Spatial Neglect”, “Motor rehabilitation for Unilateral Spatial Neglect”, “Visual rehabilitation for Unilateral Spatial Neglect”, “Sensory rehabilitation for Unilateral Spatial Neglect”, “Pharmacological intervention for Unilateral Spatial Neglect”. Particular relevance has been given to studies focusing on post-stroke USN. No publication date restrictions were applied, as seminal studies were included to provide the historical and theoretical background of the topic. Nevertheless, greater emphasis was placed on evidence published during the last decade and on original studies considered particularly relevant to the assessment and rehabilitation of USN. During data synthesis, particular attention was given to systematic reviews, meta-analyses, and randomized controlled trials whenever available, while findings from observational studies, pilot studies, and case reports were interpreted with appropriate caution according to their methodological strength.

## 3. Anatomic Correlates

USN can be caused by lesions in both hemispheres, but, as previously mentioned, it is more frequent and more severe following right-hemisphere damage. The location and extent of the lesion vary between patients [16]. Rather than resulting from damage to a single cortical region, current evidence indicates that USN reflects disruption of distributed structural and functional attention networks. Accordingly, lesions affecting cortical and subcortical regions, as well as the white matter pathways connecting them, may all contribute to the syndrome [17,18,19]. Many studies have attempted to identify the brain networks involved in USN syndrome. The inferior parietal lobe and the temporo-parietal–occipital junction have consistently been associated with USN [20,21]. With regard to the temporal lobe, another area frequently linked to USN is the superior temporal gyrus [16,22,23]. USN can also be caused by frontal lesions involving the premotor frontal areas [23] and the inferior frontal lobe [24,25]. If the lesions are confined only to the frontal lobe, the USN may be more transient. However, more commonly, large strokes affecting the territory of the middle cerebral artery involve both the critical parietal and frontal regions, leading to a severe and persistent form of USN that profoundly impacts patients’ daily functioning [4]. Referring to subcortical areas, authors found that the putamen and the caudate nucleus [26,27], the pulvinar [26], as well as the thalamus and other parts of the basal ganglia and the structures near them can be involved in the development of the syndrome [24,27]. Finally, white matter lesions affecting the connections between parietal and frontal areas have also been shown to influence the onset of neglect [17,28]. Severity and duration of USN is related to lesion extension and location, with cortical fronto-temporal areas and subcortical regions, particularly the white matter tracts connecting the posterior parietal cortex with the premotor frontal areas, as typical determinants [7].

**Figure 1:**
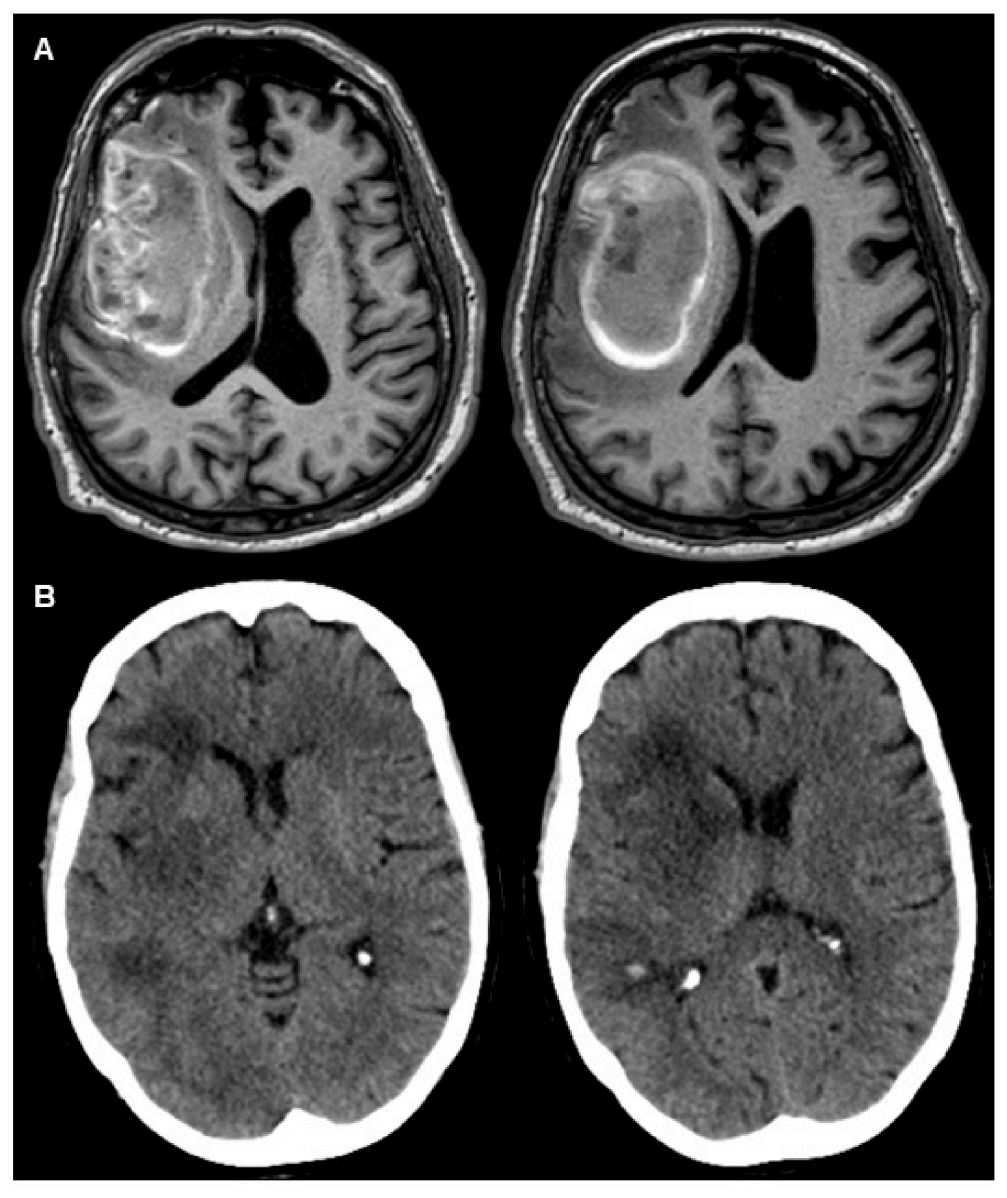
Brain imaging of two patients with unilateral spatial neglect. (**A**) Axial brain MRI of Patient 1 showing a right-hemisphere hemorrhagic lesion involving fronto-temporo-parietal regions. (**B**) Axial brain CT of Patient 2 demonstrating a right-hemisphere ischemic lesion involving fronto-insulo-temporal cortico-subcortical regions, with a small focal hemorrhagic transformation.

Figure 1 represents brain imaging of two patients with unilateral spatial neglect (USN).

## 4. Clinical Manifestations of Unilateral Spatial Neglect

In addition to standardized neuropsychological testing, USN can often be recognized through a range of observable clinical signs emerging during everyday activities. This disorder is heterogeneous, and patients may display different symptoms. In the acute phase, USN is typically associated with a deviation of the trunk, eyes, and head toward the ipsilesional side. In more severe cases, patients may fail to recognize their contralesional limbs or even the presence of people on the neglected side [4,29]. Common behaviors include bumping into objects positioned in the contralesional hemispace while walking, grooming, or attending only to the ipsilesional side of the body [3,6,9], and neglecting food placed on the contralesional side of a plate [9,30]. Patients may also show reduced attention during conversations or task performance, and in some cases, misattribute sensory stimuli from the neglected side of the body to the ipsilesional side [30].

Additional impairments variably associated with USN include neglect dyslexia and neglect dysgraphia [9,29]. In reading, patients may omit letters or words located on the left side of a page or the initial portion of single words; in writing, they may cluster words on one side, omit elements, or repeat letters within words [29]. Beyond omission-related behaviors, USN is also characterized by productive behaviors, such as perseveration on right-sided stimuli. This has been interpreted as a compensatory strategy, whereby responses in ipsilesional space are exaggerated to offset the lack of responses in contralesional space [1]. USN frequently co-occurs with other neurological conditions, including hemianopia (loss of vision in part of the visual field) and hemianaesthesia (loss of sensation in part of the body), potentially worsening their functional impact [7]. A frequent and clinically significant comorbidity is anosognosia, that is unawareness of one’s own cognitive, motor, and functional deficits, which represents a major safety risk and barrier to rehabilitation [31]. USN is also associated with deficits in processing contralesional stimuli, such as extinction and allochiria, which reflect both perceptual and attentional impairments. Extinction refers to the inability to detect a contralesional stimulus when presented simultaneously with an ipsilesional one, despite intact detection when stimuli are presented separately. This phenomenon may occur across sensory modalities (visual, auditory, somatosensory) and sometimes affects only one of them [7,31]. Allochiria, instead, refers to the mislocalization of a stimulus presented on the contralesional side, which is perceived but systematically shifted to the ipsilesional side [7].

### Subtypes of Unilateral Spatial Neglect

Given its heterogeneity, USN can be dissociated along several dimensions, including the spatial domain affected, the stage of processing involved, and the reference frame adopted [8,32].

A first dissociation concerns the type of space impacted by the disorder. Several studies have shown that distinct brain regions are involved in encoding specific spatial domains. USN can affect personal space, which refers to the space of the body itself; peripersonal space, the space within arm’s reach where interaction with objects typically occurs; and extrapersonal space, which extends beyond the range of physical interaction [33,34,35].

A second important distinction relates to the stage of processing that is impaired. USN can be categorized into perceptual, motor, and representational forms. Perceptual neglect refers to the failure to attend to or respond to stimuli located in the contralesional space, and can involve multiple sensory modalities, most commonly vision, but also audition and touch. Motor USN, on the other hand, is characterized by reduced spontaneous use or initiation of movements with the contralesional limb, despite preserved muscle strength and the absence of primary motor impairments. Closely related to this, is hypodirectional USN, a condition in which the patient, typically using the ipsilesional limb, shows reduced initiation or execution of movements directed toward the contralesional side of the space [30]. Representational neglect involves deficits in the internal representation of space. Patients with this form of neglect demonstrate impairments when mentally recalling or imagining the contralesional side of a scene or environment. For example, in a study by Bisiach and colleagues [36], patients asked to visualize familiar places (Milan Cathedral) from different imagined perspectives consistently failed to report elements located on the contralesional side, indicating that USN may affect both perceptual and mental representations of space.

A third dissociation concerns the spatial reference frame used to encode stimuli. Two main frames of reference have been identified in USN: egocentric and allocentric. In the egocentric one, spatial location is defined relative to the observer’s body, for example in reference to the head, trunk, or gaze direction. In allocentric USN, by contrast, spatial relationships are defined independently of the observer’s viewpoint, and instead depend on the spatial arrangement of objects relative to each other or within their own structure. Neurophysiological evidence suggests that different populations of neurons encode space according to these distinct frames. Moreover, lesion studies indicate that egocentric USN seems to be more frequently associated with damage to the superior parietal cortex, whereas the allocentric one appears to be more often linked to lesions in the superior temporal cortex [30].

Each of these forms of USN may occur independently or co-occur within the same individual, highlighting the syndrome’s complexity [37].

The identification of the subtype(s) of USN is important as it predicts the most impacted daily tasks and thus it may guide the choice of the best clinical intervention [32].

## 5. Theoretical Models of Unilateral Spatial Neglect

Since the 1950s and 1960s, various interpretations of the disorder have been proposed. Broadly, these can be grouped into two main categories: peripheral deficits in the sensory processing of information, and a higher-order disorder involving central processing of attention orientation and internal representation of space.

Hypotheses attributing USN to sensory processing deficits were considered in the past, but are now largely outdated. In fact, they fail to account for several key observations, such as the presence of USN in patients without sensory deficits (and vice versa), its higher prevalence after right-hemisphere lesions, and the occurrence of USN in mental imagery. The hypotheses attributing USN to a higher-level deficit began to develop from the 1970s onward. These linked the syndrome either to an impaired ability to orient attention toward the contralesional space or to a disruption of the internal representation of the contralesional hemisphere [7].

One of the first attentional theories was proposed by Kinsbourne in the 1970s [38] and was later further developed and consolidated over the years, up to 1993 [39]. According to this view, neglect reflects a functional asymmetry between two antagonistic vectors, one for each hemisphere, each directing attention toward the contralateral side of the space. While both hemispheres can orient attention contralaterally, the left hemisphere does so more forcefully. For this reason, damage to the right hemisphere leads to more severe consequences, as the right hemisphere can no longer inhibit the left hemisphere’s strong tendency to direct attention toward the right side of the space, leaving an unopposed attentional bias toward the right. Kinsbourne’s theory interprets neglect as the result of a functional asymmetry between the hemispheres.

Referring to the attentional interpretation of USN, there is also another hypothesis proposed by Heilman and colleagues [20,40]. They suggest that USN results from reduced activation of the vigilance system in the damaged hemisphere, caused by injury to the cortico-reticular activation circuit. The two hemispheres have an asymmetrical capacity for orienting attention: the left hemisphere directs attention mainly rightward, while the right hemisphere can orient attention bilaterally. Thus, when the damage occurs in the right hemisphere, there is a reduction in attention toward the left side of the space, since the left hemisphere is unable to compensate for the deficit, as it primarily directs attention to the right side of the space. This results in a strong attentional imbalance.

Posner proposed another theory [41], framing USN as a deficit in disengagement, shifting, and re-engagement of attention. In particular, he suggests that the disengagement phase is managed by the parietal lobe; therefore, parietal lesions, commonly associated with USN, would result in difficulty of shifting attention from the ipsilesional to the contralesional space. This model also supported the notion of a hemispheric asymmetry favoring the right hemisphere in attentional control.

More recently, as mentioned before (Paragraph 3), Corbetta and colleagues [42] have advanced a modern interpretation of spatial neglect as a compromission of large-scale brain networks involved in attentional control. They proposed that attentional orienting is controlled in humans by two interacting networks: a right-lateralized ventral frontoparietal network, responsible for detecting behaviorally relevant and salient stimuli and a bilateral dorsal frontoparietal network, governing goal-directed, top-down attention. According to the authors, the USN results primarily from damage to the right-lateralized ventral attention network. Damage to the ventral attention network, especially in the right temporo-parietal junction (TPJ) and ventral frontal cortex, not only impairs stimulus-driven detection but also functionally inactivate the right dorsal networks, through reduced input from the TPJ or underlying white matter damage. This model shifts the focus from localized damage to individual brain regions to a broader view of neglect as a network-level disorder.

With regard to the interpretation of the disorder as a representational deficit, Bisiach and colleagues provided evidence that USN also affects internal representations of space. In their several studies, including the Milan Cathedral experiment (described earlier in Paragraph 3.1) [36], patients failed to report details on the contralesional side of imagined scenes, suggesting that neglect extends beyond perceptual processing to mental imagery. This interpretation has been debated, as the difficulties observed could still result from an attentional deficit that prevents the subject from orienting attention to the left side of the space, regardless of whether the space is imagined or perceived [30].

Finally, given the heterogeneity of the syndrome and its different manifestations, more recent work, especially over the past three decades, has characterized USN as a multicomponent cognitive disorder. In fact, USN, can selectively affect different processes, spatial coordinates, and sensory modalities, with symptoms that may occur independently and are not necessarily present simultaneously [7].

## 6. Neuropsychological and Functional Assessment of Unilateral Spatial Neglect

Given the heterogeneity of USN, neuropsychological assessment typically involves the use of multiple tools designed to evaluate different spatial domains, reference frames, and cognitive processes. Among the affected modalities, the visual domain is the most commonly investigated [32].

When considering the spatial area involved, the evaluation of peripersonal space is often carried out using paper-and-pencil tasks aimed at detecting spatial asymmetries [37]. Commonly used tests include cancellation tasks [1,4], which can present target stimuli without other distractors, such as the Albert test [43], or with distractive stimuli, such as the Star Cancellation Test [44,45] and letter cancellation tasks [44,45,46], as well as the Bells Test [47,48] and the Apples Test [49,50]. The Apples Test is particularly useful as it allows the assessment of both egocentric and allocentric USN, by requiring patients to cancel complete and incomplete stimuli. Another test that also permits to evaluate both the coordinates of USN is the Broken Hearts test, included in the screening tool Oxford Cognitive Screen (OCS) [51,52]. In addition to quantitative scoring, cancellation tasks can also provide qualitative information of a patient’s performance, such as search strategy, exploration pattern, speed of execution, and types of errors, that may include omissions and productive manifestation [37].

Other commonly used tasks include figure copying or drawing from memory (e.g., a flower or a clock) [44,45], where patients with USN tend to omit details on the left side of the image, or they may be shifted entirely toward the right side of the space [1,4]. A common example is the clock drawing task, in which patients often omit the left half of the clock face or incorrectly place all the numbers on the right side.

Considering personal neglect, which specifically concerns the patient’s own body, it can be assessed using tasks such as the Fluff Test [53], which evaluates the ability to detect stimuli on the body and gives information about patient’s representation of the contralesional side of the body. Another test used to investigate personal neglect is the one proposed by Zoccolotti and Judica [54] and it is based on a set of everyday activities, such as combing the hair, putting on spectacles, and, depending on gender, shaving, or applying make-up. Building on these procedures, Beschin and Robertson [55] later developed the “Comb and Razor/Compact Task”, which provides a standardized way to assess USN through similar self-care tasks.

With regard to the extrapersonal space, there is no standardized procedure for its assessment and it is usually tested using an adaptation of peripersonal tasks, such as line bisection [56] or cancellation tasks, presented at a distance on large boards or screens. In fact, the assessment of extrapersonal neglect is less standardized compared to the evaluation of personal and peripersonal space. In addition, more ecological procedures have been developed, including navigation or visual search tasks in real environments, where patients are asked to detect or interact with targets distributed across a wider space, or description of rooms task [57]. Recently, the Visual Scanning Test [58] has been developed as a standardized tool for assessing extrapersonal neglect and it consists of a visual search task.

Some of these afore-mentioned tasks are included in standardized battery [44,45,59]. The Behavioral Inattention Test (BIT) is one of the most used to assess USN and combines six conventional paper and pencil tasks with nine more ecologically behavioral tasks. Other ecological tasks to evaluate USN in contexts similar to activities of daily living are included in the set of tasks proposed by Zoccolotti and Judica [54].

In addition to neuropsychological tests, functional impact of USN is another important aspect to assess. Catherine Bergego Scale (CBS) [60,61] evaluates USN in everyday activities across different spatial domains. To increase scoring consistency, the Kessler Foundation Neglect Assessment Process (KF-NAP) has further improved its standardization and reliability [8,11]. In a recent work, Williams and colleagues [62] show its high validity and recommend it for the assessment of functional aspects of USN.

In addition to the assessment of neglect across different spatial domains, several instruments have been developed to investigate other manifestations of the syndrome. Among these, neglect dyslexia has received particular attention, as it reflects the impact of spatial attentional deficits on reading processes. It can be assessed using reading tasks [59,63], where patients with left USN generally show omissions, and substitutions in the left half of the sentence or words [64].

Although traditional assessment of USN relies mainly on clinical observation and traditional paper-and-pencil neuropsychological tools, since the late 1990s, digital tools, including computer-based, touchscreen, and virtual reality (VR) technologies, have increasingly been introduced to support and enhance diagnostic procedures [37]. These technologies can offer greater sensitivity, diagnostic accuracy, improved psychometric properties, particularly diagnostic validity, as well as reduced costs and shorter administration times [65,66,67,68]. One of the main advantages of these technologies is their ability to simulate real-life contexts, allowing for a more ecological assessment of patients’ behavior. In addition, their digital nature enables remote administration and facilitates the collection of richer and more diverse data, which may be critical for characterizing the severity and specific profile of neglect [37,68].

Over the past five years, several studies have focused on the integration of VR-based tools for USN assessment, either by digitizing traditional tests or designing novel paradigms. Advances in immersive VR have made it possible to create highly realistic environments, enabling more natural interactions, thus enhancing ecological validity [69]. Among non-immersive VR setups, the Virtual Reality Lateralized Attention Test (VRLAT) has shown good reliability and validity in detecting spatial neglect, with the ability to identify subtle deficits not captured by traditional paper-and-pencil tests, likely due to its higher ecological validity [70].

Finally, among emerging technology-based assessment approaches, eye tracking has attracted increasing interest as a complementary tool for the objective evaluation of visuospatial exploration in patients with USN. A recent review of Tomaselli and colleagues [71] identified a characteristic visual search profile, including reduced saccade amplitudes, a restricted exploration field and a rightward shift of visual fixations, suggesting that eye tracking may improve the detection of subtle USN deficits. Moreover, eye-tracking measures may provide additional information on individual exploration patterns that could potentially support the development of targeted rehabilitation approaches. A recent scoping review [72] highlighted the growing interest in eye tracking for the objective evaluation of visual exploration deficits, while also emphasizing the considerable heterogeneity of assessment protocols and the need for further standardization before routine clinical implementation.

Despite these promising advantages, no digital tool has yet reached the status of a gold standard for USN assessment, mainly due to the lack of normative data and large-scale validation studies. Consequently, while digital and VR tools are emerging as valuable complements to clinical practice, standardized paper-and-pencil tests continue to represent the reference standard in the diagnosis of spatial neglect [37].

In Table 1, the main assessment tools are reported.

## 7. Non-Pharmacological Rehabilitation Approaches for Unilateral Spatial Neglect

Given the high incidence of USN in the acute phase after stroke and its negative impact on other deficits such as hemiplegia and daily functional activities, it is crucial to address this syndrome through targeted rehabilitation. Over the years, a wide range of rehabilitation techniques for USN have been proposed. Rehabilitation approaches for USN have traditionally been broadly described as top-down or bottom-up interventions. Top-down approaches involve active engagement of the patient’s attentional resources, encouraging voluntary orientation of attention toward the neglected hemispace, while bottom-up approaches rely on passive sensory or visuomotor stimulations and induce an implicit reorientation of spatial attention toward the neglected side. Early theoretical and clinical work on attention and neglect has described interventions consistent with this distinction [73,74]. However, this distinction is increasingly considered an oversimplification, as many interventions involve overlapping mechanisms, as reflected in more recent classification [75]. This narrative review focuses on the most clinically relevant and commonly discussed approaches, according to their clinical characteristics.

Among visual and cognitive interventions, one of the most used and recommended rehabilitation treatment in clinical practice is visual scanning training (VST), that consists of training patients to adopt systematic visual exploration strategies of the neglected hemispace. Training usually involves tasks like visual search, cancellation tasks, copying tasks, and reading exercises, with progressively increasing complexity. VST is typically supported by the use of external cues and feedback. Visual or verbal prompts are frequently employed to guide attention towards the neglected hemispace, while continuous feedback helps reinforce correct exploratory behaviors and supports learning processes [76,77]. Early evidence supported VST in a randomized controlled trial [78] and was subsequently reinforced by a prospective uncontrolled study [79], both reporting improvements in reading and visuospatial performance in patients with right-hemisphere damage, with some evidence of generalization to ecological situations. High-level evidence from a recent systematic review and meta-analysis of RCTs showed that VST can improve performance on visuospatial tasks and neglect-specific assessments [80]. However, the more recent Cochrane systematic review of 2021 [75] has highlighted that the overall quality of evidence remains low with heterogeneous results, suggesting that VST is not clearly superior to other rehabilitation approaches, with its effectiveness likely depending on patient characteristics and timing of intervention [81].

Another cognitive intervention involves the use of explicit strategies aimed at enhancing patients’ awareness and voluntary control of attention. These include systematic exploration routines and strategy-based approaches that encourage patients to actively compensate for their deficits and evidence suggests that these approaches can improve performance on structured tasks and neglect-specific assessments, evidenced both in a non-systematic review of Kerkhoff et al. [77] and in the Cochrane systematic review of 2013 [76]. However, their effectiveness may depend on the patient’s cognitive abilities and level of awareness.

Another approach that is frequently investigated and applied in clinical practice is prism adaptation (PA). This procedure involves wearing prismatic lenses that shift the visual field toward the ipsilesional side, inducing a visuomotor recalibration reflected by a compensatory after-effect toward the neglected hemispace [82,83,84]. This shift is considered a behavioral marker of sensorimotor adaptation and is thought to reflect a partial realignment of visuomotor coordinates that can contribute to the rehabilitation of spatial neglect [85]. Evidence supporting prism adaptation comes from early controlled clinical studies [82], a randomized controlled trial [83], and prospective interventional studies [84]. These studies have investigated the effects of both a single prism adaptation session [82,84] and repeated sessions [83] on USN, consistently reporting improvements in neuropsychological test performance. Prism adaptation has also been shown to influence the tactile modality in patients with USN and tactile extinction [86]. On the other hand, findings are inconsistent considering the impact of PA on allocentric deficits, as has emerged in a recent case study [87] and in a prospective uncontrolled interventional study [88].

A Cochrane systematic review and several systematic reviews with meta-analyses, including meta-analyses restricted to randomized controlled trials (RCTs) [75,89,90,91], reported heterogeneous results regarding the effectiveness of this treatment, suggesting that PA may produce short-term improvements that are not maintained over the long term [89,91]. Overall, the effectiveness of PA appears to depend on treatment parameters, such as prism deviation and session intensity, and all the recent reviews highlight the need for standardized protocols and higher-quality clinical trials.

Among approaches that involve body awareness, mirror therapy has emerged as a promising bottom-up intervention for USN after stroke. By using the reflection of the unaffected limb to create the illusion of movement in the neglected hemispace, mirror therapy is thought to engage visuomotor integration and promote attentional reorientation toward the contralesional side. Evidence from a recent systematic review suggests improvements in neglect-specific tasks, although results of its efficacy are heterogeneous and limited by small sample sizes [92]. Overall, it appears to be a promising and accessible intervention that may be used within multimodal rehabilitation programs. Neck muscle vibration is another body-based approach that modulates proprioceptive input and egocentric spatial representations. An early prospective experimental within-subject study showed that vibration of the posterior neck muscles can temporarily reduce contralesional neglect and the pathological rightward bias, by shifting the perceived trunk midline [93]. In a subsequent study, the same group further showed that neck proprioceptive and vestibular inputs influence ocular exploration of space, suggesting that modulation of these may facilitate exploration of the neglected hemispace [94]. In a more recent randomized controlled trial, Choi and collaborators [95] have found an improvement in peripersonal neglect when neck vibration was combined with PA; considering the functional abilities, an improvement in CBS scores was also found in patients with USN, but this improvement was also observed when each intervention was applied individually.

Among movement-based approaches, limb activation is a commonly used intervention based on the idea that activating the contralesional limb can help reorient attention toward the neglected side. This can be achieved through either active movements or passive stimulation, such as proprioceptive input. An early single-case experimental study showed that limb activation can transiently improve performance in spatial tasks, likely by enhancing exploratory motor behavior rather than directly restoring perceptual deficits [96], also through passive limb movements, as has emerged some years later in a within-subject experimental study [97]. However, these effects are generally short-lived and variable across patients, limiting its use as a standalone treatment. For this reason, it is often used in combination with other rehabilitation approaches, such as visual scanning training or prism adaptation [76,98]. Despite these limitations, it remains a practical and low-cost adjunct that can be easily implemented in clinical settings.

Non-invasive brain stimulation techniques, including transcranial direct current stimulation (tDCS) and transcranial magnetic stimulation (TMS), have been increasingly investigated for the rehabilitation of USN after stroke. Some systematic reviews and meta-analysis published in the last decade suggest that these approaches can produce modest improvements in neglect severity, particularly on neuropsychological tests [99,100,101]. Across studies, the most commonly adopted tDCS montage is interhemispheric, with anodal stimulation over the lesioned hemisphere and cathodal stimulation over the contralesional hemisphere, as well as inhibitory low-frequency TMS applied to the intact hemisphere. However, a consistent limitation that emerges across the more recent systematic reviews and meta-analysis is the substantial heterogeneity in stimulation protocols and study design, which complicates the identification of optimal parameters [99,100,101,102,103]. Some evidence suggests that inhibitory TMS protocols applied to the contralesional hemisphere may yield more consistent effects compared to tDCS, although findings remain heterogeneous [101]. Overall, these techniques are generally used in combination with other rehabilitation approaches, given the variability of results and the limited evidence on functional outcomes.

Among electrical stimulation approaches, transcutaneous electrical nerve stimulation (TENS) has been proposed as a technique to modulate spatial attention through somatosensory input. Early experimental studies showed that TENS applied to the left side of the neck can transiently reduce neglect symptoms [104], and improve performances on tasks of mental representations of objects and space [105], as well as postural control [106]. A more recent single-case experimental study has investigated its use in combination with other interventions, suggesting a potential role of a combined training (ecological VST and TENS) on the activities of daily living [107].

Among sensory stimulation approaches, historically defined as bottom-up interventions, techniques such as eye patching, optokinetic stimulation, and vestibular stimulation have been proposed to modulate spatial attention through visual or vestibular input. Eye patching can be carried out by monocular or hemifield patching and has been proposed as a technique to rebalance interhemispheric activation and modulate visuomotor orienting responses via the superior colliculus pathway [1,108,109]. An early prospective randomized controlled study [108] and descriptive interventional study [110] have shown some improvements on neuropsychological tests, although evidence remains inconsistent, depending on methodological parameters (monocular vs. hemifield occlusion, lesion site, poststroke stage), and its long-term functional benefits have not been clearly demonstrated as effects on functional outcomes are unclear, as emerged in a systematic review and meta-analysis on rehabilitative approaches for USN and in a systematic literature review on eye patching [80,109]. Optokinetic stimulation is carried out by using moving visual stimuli (black dots, lines) to enhance visual attentional shift toward the neglected space, by eliciting a nystagmus [77]. It has been associated with short-term improvements in visuospatial exploration most evident on paper-and-pencil tasks, also when combined with conventional rehabilitation [111]. However, a recent systematic review shows that its effects are often task specific, depending on the intensity of the treatment and the combination with other approaches [112]. Vestibular stimulation can be caloric (introduction of water in the external ear canal) or galvanic (direct electrical stimulation of the vestibular nerves). These techniques can transiently improve USN symptoms by modulating the activity of the peripheral vestibular system, altering vestibular afferent signaling and transiently influencing the vestibulo-ocular reflex and spatial orientation. Some experimental studies [113,114] suggest that caloric stimulation appears to produce more consistent short-term effects, especially when applied to the contralesional ear and this has been further supported in a recent systematic review on this topic [115]. Concerning the caloric stimulation, transient improvements in motor deficits have also been reported in an experimental single-case report and subsequently supported by a small controlled study [116,117]. Regarding galvanic vestibular stimulation, a recent systematic review and individual experimental studies have reported variable findings, and the overall evidence remains limited and heterogeneous [115,118,119,120]. Overall, these approaches may induce transient improvements in spatial attention, but their clinical applicability is limited by methodological variability, inconsistent findings, and uncertain long-term benefits.

Technology-based interventions, including virtual reality (VR), computer-based training, and robotic-assisted rehabilitation, are increasingly explored in the management of USN after stroke. VR, in particular, offers the possibility to create controlled, ecologically valid environments that can enhance spatial exploration through multisensory feedback and task engagement [69,121]. Recent systematic reviews suggest that both immersive [121,122] and non-immersive VR [121] approaches may improve USN-related visuospatial performance, although findings remains variable across studies and dependent on protocol characteristics. Beyond USN rehabilitation, findings from a randomized controlled trial suggest that immersive VR may have broader applications in post-stroke cognitive rehabilitation [123]. However, evidence is still limited by small sample sizes, heterogeneity of interventions, and lack of standardized outcomes [69,121]. VR-based interventions often incorporate elements of traditional rehabilitation approaches, such as visual scanning training, within more ecological and interactive environments [69,124]. Computer-based cognitive rehabilitation programs similarly aim to enhance visuospatial exploration through structured tasks, with some evidence of benefit on neglect-related measures, evidenced in a systematic review conducted by Svaerke et al. [125]. Concerning robotic-assisted training, combining motor and spatial components, a systematic review conducted by Bazan et al. [126] has shown promising but still preliminary positive effects. Emerging approaches such as brain–computer interfaces (BCI) aim to directly modulate attentional networks through real-time feedback, but remain at an early, exploratory stage [127].

Artificial intelligence (AI) has attracted increasing interest in neurorehabilitation in recent years, particularly as a tool to enhance the personalization and efficiency of technology-assisted interventions. A recent systematic review [128] examining AI applications in stroke and neurological rehabilitation highlighted its potential to improve treatment delivery through adaptive task progression, automated performance monitoring, and real-time feedback. AI-based systems can be integrated into virtual reality platforms, robotic devices, and computer-based rehabilitation programs, allowing interventions to be tailored to individual patient performance and promoting greater patient engagement. However, despite these promising developments, evidence specifically addressing USN remains scarce, and further well-designed clinical studies are required before AI-driven approaches can be routinely implemented in clinical practice. Overall, these technology-driven interventions appear promising, particularly due to their flexibility, scalability, and ability to integrate multisensory stimulation. Nevertheless, current evidence remains limited by methodological heterogeneity.

Despite the wide range of rehabilitation approaches available, systematic reviews and evidence syntheses indicate that improvements are more consistently observed on standardized neuropsychological tests, whereas evidence for sustained functional benefits and improvements in activities of daily living remains limited and heterogeneous [75,89,112]. Current clinical guidelines similarly highlight the uncertainty regarding long-term functional outcomes [129].

Overall, the available evidence is heterogeneous across rehabilitation approaches. The strongest evidence is currently available for interventions such as visual scanning training and prism adaptation, which have been evaluated in several systematic reviews, although the certainty of evidence remains low to moderate because of methodological heterogeneity. For many other interventions, including mirror therapy, limb activation, sensory stimulation techniques, and technology-based rehabilitation, the evidence is mainly derived from small randomized trials, highlighting the need for larger, well-designed clinical trials using standardized outcome measures.

In Table 2, the main non-pharmacological rehabilitation approaches are reported.

## 8. Pharmacological Intervention for Unilateral Spatial Neglect

Pharmacological interventions have also been investigated as potential adjunctive strategies for the treatment of USN, based on the involvement of different neurotransmitter systems in attentional and cognitive processes. In particular, pharmacological strategies targeting cholinergic, dopaminergic, and noradrenergic pathways have been explored. However, evidence supporting their clinical efficacy remains limited. A Cochrane systematic review found insufficient evidence to establish the effectiveness of pharmacological interventions for USN after stroke, mainly due to the small number of available studies and their methodological limitations [130]. Similarly, a systematic review by Van der Kemp et al. [131] highlighted some preliminary beneficial effects of pharmacological modulation, particularly with cholinergic modulation, but concluded that current findings remain inconclusive and require confirmation in larger, well-designed clinical trials. Although pharmacological approaches have been explored as potential adjunctive treatments, they remain poorly represented in the rehabilitation literature. A recent systematic review of randomized controlled trials similarly showed that pharmacological interventions remain underrepresented within the rehabilitation literature for USN, highlighting the need for further investigation [132].

## 9. Clinical Case Presentation

The patient is a 79-year-old woman with 11 years of formal education, retired who had previously worked as a skilled office employee. She was admitted to the Stroke Unit after an ischemic stroke that involved the right middle cerebral artery territory, with lesions in the right fronto-insulo-temporal cortico-subcortical regions extending into the striato-capsular one, with a small focal hemorrhagic transformation (see Figure 1B).

The patient had a CRI-Q score [133] of 98, indicating a medium level of cognitive reserve, and an IQCODE score [134] of 3, suggesting no evidence of prior cognitive impairment. She was fully independent in activities of daily living before the stroke. On admission to the ward, the NIHSS score [135] was 19: she was drowsy but easily arousable, with dysarthric speech and inconsistent responses to questions. Neurological examination revealed rightward gaze deviation, severe left-sided neglect, left hemiplegia, and left hemisensory loss with extinction to double tactile simultaneous stimulation. No blink to visual threat was observed in the left hemifield.

**Figure 2:**
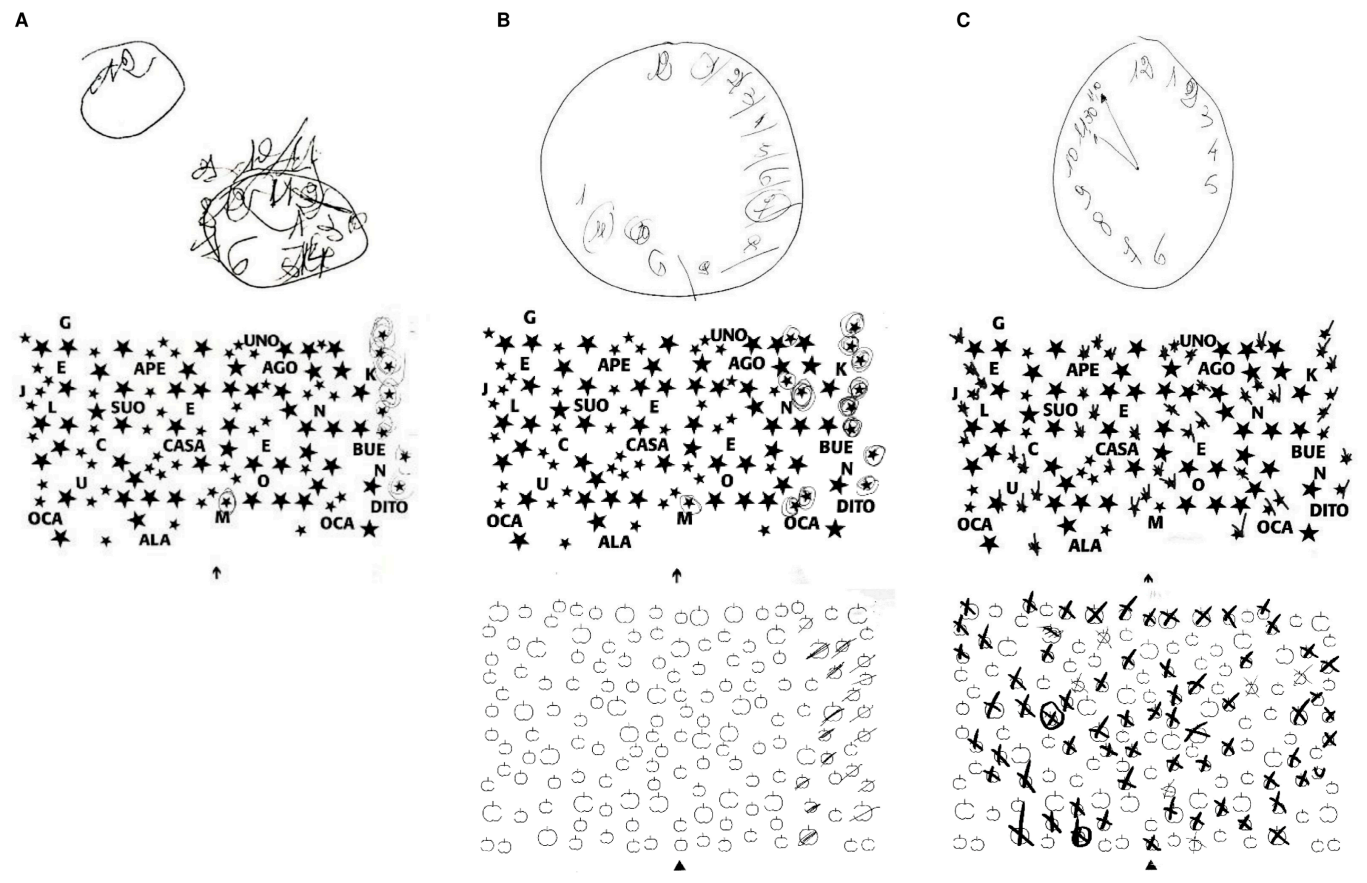
Representative examples of the neuropsychological tests administered at the three neuropsychological assessments. (**A**) Clock Drawing Test and Star Cancellation at the first evaluation (T0) showing a strong rightward attentional bias, a severe spatial disorganization, and marked left-sided omissions. (**B**) Clock Drawing Test, Star Cancellation Test, and Apples Test at T1 showing persistent spatial disorganization with clustering of elements in the ipsilesional hemispace and ongoing left-sided omissions with slight improvement in exploration and the presence of both egocentric and allocentric neglect. (**C**) Clock Drawing Test, Star Cancellation Test, and Apples Test at T2 showing improved exploration of the left space with reduced omissions but residual spatial disorganization and persistent allocentric errors. The arrow indicates the correct orientation of the test sheet.

The first neuropsychological evaluation was carried out 5 days after the stroke onset (T0). The patient was partially oriented in space and time, aware of the occurred cerebrovascular event but anosognosic for cognitive, motor, and functional deficits occurring after stroke. As the patient showed marked fatigability, the initial neuropsychological assessment was limited to the MoCA Test [136,137], the Clock Drawing Test [138], and the Star Cancellation Test of the BIT [44,45] (see detailed scores on Table 3). The MoCA Test [136,137] suggested a global cognitive impairment, particularly affecting the visuo-spatial, attention, and orientation domains; however, results were interpreted with caution given the presence of marked USN. The performance on the Clock Drawing Test [138] was impaired, with difficulty in mental representation of the object and organization of the elements in the space. The Star Cancellation Test [44,45] confirmed the severe left peripersonal USN, with marked rightward bias (Figure 2A).

One week later, the patient underwent a second neuropsychological evaluation (T1). She presented with less degree of fatigability and an improvement on cooperation. She was fully oriented in space but remained partially oriented to time, with errors in reporting the day of the month. She still showed a rightward gaze preference, although she was able to cross the midline if stimulated. Detection of single visual stimuli in the left hemifield was inconsistent, whereas performance in the right hemifield was preserved, likely due to the presence of a left homonymous hemianopia. Fluctuations were also observed during double simultaneous tactile stimulation. An increased awareness of cognitive, functional, and motor deficits was noted, consistent with emergent awareness according to Crosson’s classification of awareness [139]. In this assessment, the patient underwent a more extended neuropsychological assessment, including some conventional and behavioral BIT [44,45] subtests, the Apple Test [49,50], a reading task [59] and the Clock Drawing Test [138] (see detailed scores on Table 3). The CBS [60,61] was also administered. Results confirmed the persistence of a severe left peripersonal USN, involving both egocentric and allocentric components, as well as neglect dyslexia. Compared to the initial assessment on the Star Cancellation task [44,45], the patient showed only minimal improvement on the detection of target stimulus with performance still markedly impaired (Figure 2B). Qualitative assessment also revealed extrapersonal USN on a room description task, with omission of elements presented on the left side of the space. The CBS score [60,61] suggested a mild level of functional neglect, based on the administered items; in fact, several items could not be assessed, as the patient remained mostly bedridden and was unable to walk and perform many daily activities. Left-sided hemiplegia persisted, which was more pronounced in the upper limb.

Three days later, the patient was transferred to a rehabilitation clinic where she underwent approximately 1.5 months of multidisciplinary rehabilitation, including cognitive, motor, and functional therapy. According to the available clinical information, the rehabilitation program included visual scanning training combined with awareness training to improve exploration of the left hemispace. During the later stages of rehabilitation, these interventions were complemented by training targeting sustained, selective, and shifting attention, as well as executive functions. Detailed information regarding the frequency, duration, intensity, and specific organization of the rehabilitation program was not available, as the rehabilitation was conducted at an external institution.

The last neuropsychological follow-up was carried out at the discharge from the rehabilitation center (T2). The patient has undergone the same tests as the prior assessments and she has shown an overall improvement in cognitive abilities and left-sided exploration (see detailed scores on Table 3). Tests assessing peripersonal USN indicated improvement in egocentric neglect and neglect dyslexia, while allocentric neglect was still present (Figure 2C). Performance on some tests remained below the normal range, also influenced by the patient’s difficulty on sustained attention. Considering the functional perspective, mild USN was still present, with only slight changes in the domains assessed by the CBS [60,61]. The patient also improved the exploration of the left extrapersonal and personal space, although a residual mild rightward bias remained. Awareness further increased, reaching a level consistent with anticipatory awareness according to Crosson’s model [139].

## 10. Limitations of the Current Evidence

The available evidence on USN is limited by substantial methodological and clinical heterogeneity. Studies differ considerably in terms of patient characteristics, time since stroke, and the instruments used to identify and quantify neglect. A wide range of paper-and-pencil tasks, functional scales, experimental paradigms, and technology-based tools have been employed, making comparisons across studies difficult. Assessment of some spatial domains, particularly extrapersonal neglect, remains less standardized, while emerging tools such as virtual reality and eye tracking still lack sufficiently large normative datasets and extensive clinical validation.

Similar limitations characterize the rehabilitation literature. Intervention protocols vary widely with regard to treatment modality, intensity, duration, stimulation parameters, outcome measures, and follow-up periods. Many studies include relatively small and clinically heterogeneous samples, which limits statistical power and the generalizability of their findings. In addition, only a limited number of adequately powered, high-quality randomized controlled trials are available for most rehabilitation approaches.

Another important issue is that treatment-related improvements are more consistently reported on neglect-specific neuropsychological measures than on functional outcomes. Evidence of sustained benefits in activities of daily living, independence, and everyday participation remains limited and heterogeneous. This distinction is particularly relevant because improvements on structured tasks do not necessarily translate into meaningful changes in daily functioning.

Overall, although several interventions, including visual scanning training and prism adaptation, have shown encouraging results, the current evidence does not allow firm conclusions regarding the comparative effectiveness of different rehabilitation approaches. Future studies should use more standardized diagnostic criteria, validated and clinically meaningful outcome measures, clearly described treatment protocols, adequately powered samples, and longer follow-up periods. Greater methodological consistency would improve comparability across studies and help determine which interventions provide sustained and functionally relevant benefits.

## 11. Conclusions

Unilateral spatial neglect (USN) is a complex and often disabling consequence of stroke. It is commonly associated with right-hemisphere lesions and its clinical presentation is highly variable and may involve different spatial domains, sensory modalities, and reference frames. For this reason, USN should not be considered a single symptom, but rather a heterogeneous syndrome that can interfere with many aspects of daily life and rehabilitation.

A comprehensive assessment is essential to identify both the presence and the specific profile of USN. Although traditional paper-and-pencil tests remain useful, they do not fully capture the impact of the disorder in everyday activities. For this reason, functional scales and ecological observations should therefore be included. As illustrated by the clinical case presented in this review, repeated assessments may also be valuable for monitoring changes in USN and in the broader cognitive profile over time.

Several rehabilitation approaches are available, but the evidence remains variable and no single treatment can be considered a gold standard. Management of USN should be individualized according to patient-specific characteristics, including the type and severity of USN, lesion futures, associated cognitive, motor and functional deficits, awareness of the disorder, and stage of recovery. Rehabilitation strategies should therefore be selected and adapted within a comprehensive, multidisciplinary program rather than applied according to a uniform approach. Early recognition and targeted intervention remain essential to support functional recovery and independence after stroke. Further high-quality clinical trials are needed to support the development of more standardized and evidence-based rehabilitation protocols.

**Figure 3:**
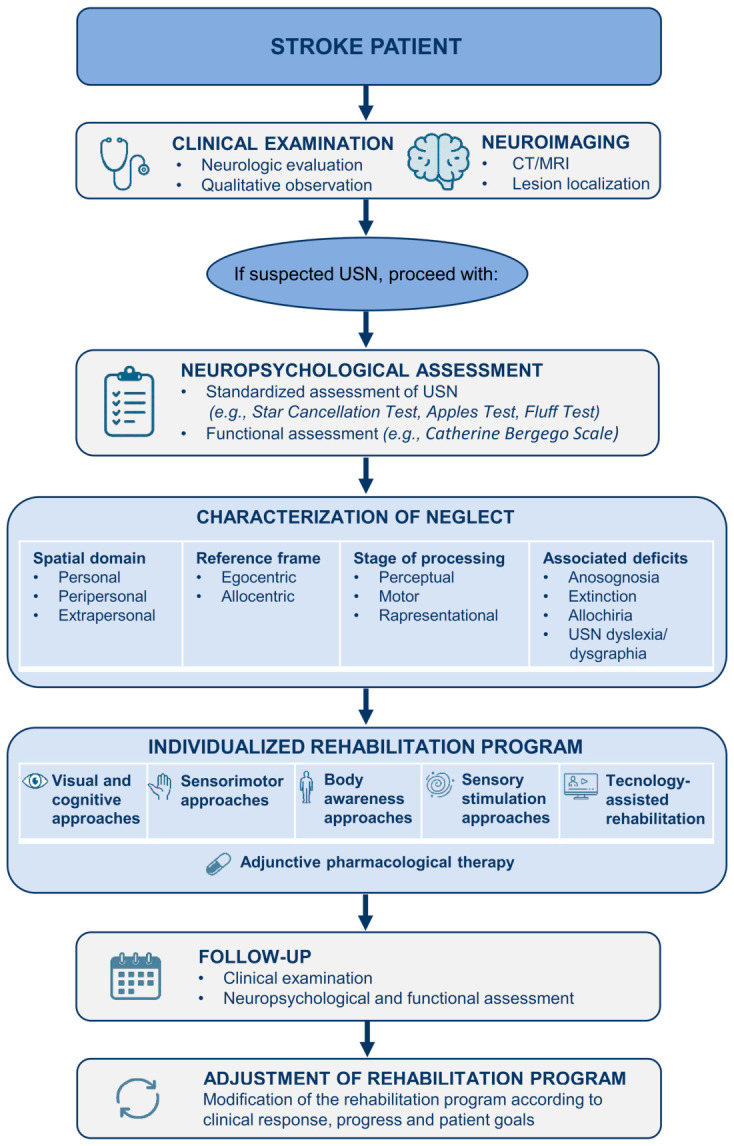
Proposed clinical pathway for the assessment and management of post-stroke unilateral spatial neglect (USN).

Based on the clinical and therapeutic aspects discussed above, we propose a practical pathway for the assessment, management, and longitudinal reassessment of patients with post-stroke USN (Figure 3).

## Figures and Tables

**Table 1:** Summary of the main assessment tools used to evaluate unilateral spatial neglect (USN) across spatial domains.

Tested Space	Task Type	Brief Description and Citations
Peripersonal space	Cancellation tasks	Patients have to cancel target stimuli with or without distractive stimuli Line Cancellation [43]; Star Cancellation [44,45]; Letter Cancellation [44,45,46]; Bells Cancellation [47,48]; Apples Test [49,50]; Broken Hearts [51,52]
	Copying tasks	Tasks where patient have to copy standard figures [44,45]
	Drawing from memory	Reproduction of familiar figures from memory [44,45]
Personal space	Fluff Test	Patient is asked to remove cardboard circles attached to their body [53].
	Comb and Razor/Compact Test	Patient simulates body-centered exploration tasks (combing hair or shaving/applying powder) [55]
	Zoccolotti and Judica tasks	Patient simulates body-centered exploration tasks (combing the hair, putting on spectacles, and, depending on gender, shaving or applying make-up) [54]
Extrapersonal space	Distant cancellation/line bisection	Classical cancellation or bisection tasks performed on a board placed at some distance Visual Scanning Test [58]
	Room description	Patient describes what they see in a room
	Environmental visual search	Functional search for objects in real or simulated environments
Neglect dyslexia	Sentence reading task [59,63]	Patients read sentences; omissions or substitutions of words/letters on the contralesional side are recorded
Functional assessment	Catherine Bergego Scale (CBS)	Observational scale evaluating neglect in everyday activities [60,61]
	Zoccolotti and Judica [54] functional tasks	Ecological tasks to assess neglect in daily life situations
Technology-based/ecological assessment	Virtual reality tasks; Eye-tracking paradigms	Evaluation of spatial exploration in more naturalistic environments (Virtual Reality Lateralized Attention Test; VRLAT) [70]; quantitative analysis of gaze behavior during visual search tasks
Comprehensive batteries	BIT—Behavioral Inattention Test	Standardized battery combining paper-and-pencil tests (cancellation, line bisection, figure copying, etc.) with behavioral subtests [44,45]
	Battery for Attentional Disorders	Italian battery including tasks for attention and neglect assessment [59]

**Table 2:** Non-pharmacological rehabilitation approaches for unilateral spatial neglect (USN): current evidence, clinical benefits, and limitations.

Rehabilitation Approach	Current Level of Evidence	Main Clinical Benefits	Main Limitations
Visual scanning training (VST)	Low–moderate evidence from RCTs, systematic reviews and Cochrane analyses	Improves performance on visuospatial tasks and USN-specific assessments; possible generalization to ecological activities	Evidence quality remains limited; heterogeneous results; unclear superiority compared with other interventions; functional generalization is inconsistent
Strategy-based cognitive rehabilitation/awareness training	Low–moderate evidence from systematic reviews and clinical studies	May improve compensatory strategies, awareness of deficits, and performance on structured tasks	Effectiveness depends on cognitive abilities and awareness level
Prism adaptation (PA)	Low–moderate evidence from controlled studies, RCTs, systematic reviews and meta-analyses	Short-term improvement in USN symptoms and visuospatial tasks; inconsistent findings for its impact on allocentric deficits and for generalization to ecological activities	Results are heterogeneous; long-term maintenance remains uncertain; effects depend on treatment parameters and standardized protocols are lacking
Body-awareness approaches (mirror therapy)	Preliminary evidence from systematic reviews and small clinical studies	Possible improvement in USN-specific tasks through visuomotor integration and attentional reorientation; may be useful as an adjunctive intervention	Limited number of studies; small sample sizes; heterogeneous results of its efficacy
Proprioceptive stimulation (neck muscle vibration)	Preliminary evidence from experimental studies and small clinical trials	Transient improvement in spatial exploration and reduction of rightward bias; may be useful as an adjunctive intervention	Effects are often short-lived; limited evidence as standalone treatment; functional benefits remain unclear
Movement-based approaches (limb activation)	Preliminary evidence from experimental studies	Transient improvement in spatial exploration; may be useful as an adjunctive intervention	Effects are often short-lived; variability between patients; limited evidence as standalone intervention
Non-invasive brain stimulation (TMS/tDCS)	Low–moderate evidence from systematic reviews and meta-analyses	Modest improvements in USN severity, mainly on neuropsychological tasks	High heterogeneity in stimulation protocols; optimal parameters unclear; limited evidence for functional outcomes
Electrical stimulation (TENS)	Low/preliminary evidence from experimental studies	Transient reduction of USN symptoms and improvement on postural control; potential adjunctive effects when combined with conventional rehabilitation	Effects on USN symptoms are often transient
Sensory stimulation (eye patching, optokinetic stimulation, vestibular stimulation)	Low/preliminary evidence from experimental studies, RCTs and systematic reviews	Transient improvements in spatial attention, visuospatial exploration and neuropsychological tasks; caloric vestibular stimulation appears to produce more consistent short-term effects compared with other sensory approaches	Effects are mainly short-term; findings are heterogeneous and often task specific; methodological variability; limited evidence for sustained functional benefits
Technology-based rehabilitation (virtual reality, computer-based training, robotic-assisted rehabilitation, BCI, artificial intelligence)	Preliminary to low–moderate evidence from systematic reviews and experimental studies	More ecological and engaging training environments; improvements mainly observed on USN-related neuropsychological measures	Small samples, heterogeneous systems and protocols; lack of standardized outcomes; limited evidence for functional transfer

Abbreviations: USN, Unilateral spatial neglect; RCTs, Randomized controlled trials; TMS, Transcranial magnetic stimulation; tDCS, Transcranial direct current stimulation; TENS, Transcutaneous electrical nerve stimulation; BCI, Brain–computer interface.

**Table 3:** Raw, adjusted, and equivalent scores (ES) and cutoffs (CO) in the three neuropsychological assessments (T0, T1, T2).

	T0			T1			T2		
	Raw Score	Adjusted Score	Equivalent Score/Cut-Off	Raw Score	Adjusted Score	Equivalent Score/Cut-Off	Raw Score	Adjusted Score	Equivalent Score/Cut-Off
MoCA	16	16.75	ES 0 *				21	21.75	ES 2
Clock Drawing Test	0	0	ES 0 *	4	4	ES 0 *	10	10	ES 2
BIT conventional subtests									
Star Cancellation Test	8*		CO 51	13*		CO 51	44*		CO 51
Line Cancellation Test				18*		CO 34	36		CO 34
Line Bisection Test				4*		CO 7	5*		CO 7
Figure Copying				0*		CO 2	3		CO 2
BIT behavioral subtests									
Picture description task				0*		CO 5	4*		CO 5
Apple test									
Number of complete apples				8*		CO 45	48		CO 45
Egocentric asymmetry score (complete apples)				8*		CO 2	2		CO 2
Allocentric asymmetry score (incomplete apples)				10*		CO 1	15*		CO 1
Sentence reading task				4*		CO 6	6		CO 6
CBS KF-NAP score				8		Mild *	6.6		Mild *

* Indicates a patient score within the pathological range. Abbreviations: MoCA, Montreal Cognitive Assessment; BIT, Behavioral Inattention Test; CBS, Catherine Bergego Scale; KF-NAP, Kessler Foundation Neglect Assessment Process.

## Data Availability

The original contributions presented in this study are included in the article. Further inquiries can be directed to the corresponding author.

## Abbreviations

The following abbreviations are used in this manuscript:

USN	Unilateral Spatial Neglect
CBS	Catherine Bergego Scale
KF-NAP	Kessler Foundation Neglect Assessment Process
BIT	Behavioral Inattention Test
MoCA	Montreal Cognitive Assessment
NIHSS	National Institutes of Health Stroke Scale
IQCODE	Informant Questionnaire on Cognitive Decline in the Elderly
CRI-Q	Cognitive Reserve Index questionnaire
OCS	Oxford Cognitive Screen
VR	Virtual Reality
VRLAT	Virtual Reality Lateralized Attention Test
BCI	Brain-Computer Interface
VST	Visual Scanning Training
PA	Prism Adaptation
tDCS	Transcranial Direct Current Stimulation
TMS	Transcranial Magnetic Stimulation
TENS	Transcutaneous Electrical Nerve Stimulation
RCTs	Randomized controlled trials
